# Chitosan Nano/Microformulations for Antimicrobial Protection of Leather with a Potential Impact in Tanning Industry

**DOI:** 10.3390/ma15051750

**Published:** 2022-02-25

**Authors:** David S. Freitas, Pilar Teixeira, Inês B. Pinheiro, Elisabete M. S. Castanheira, Paulo J. G. Coutinho, Maria J. Alves

**Affiliations:** 1 Centre of Chemistry and Department of Chemistry, University of Minho, Campus de Gualtar, 4710-057 Braga, Portugal; davidsfreitas@ceb.uminho.pt (D.S.F.); inespinheiro@bio.uminho.pt (I.B.P.); 2 CEB—Centre of Biological Engineering, University of Minho, Campus de Gualtar, 4710-057 Braga, Portugal; pilar@ceb.uminho.pt; 3 LABBELS—Associate Laboratory, 4800-122 Braga, Portugal; 4 Centre of Physics of Minho and Porto Universities (CF-UM-UP), University of Minho, Campus de Gualtar, 4710-057 Braga, Portugal; ecoutinho@fisica.uminho.pt; 5 LaPMET—Laboratory of Physics for Materials and Emergent Technologies, 4800-122 Braga, Portugal

**Keywords:** chitosan nanostructures, silver nanoparticles, antimicrobial activity, leather, tanning industry

## Abstract

Tanned leather can be attacked by microorganisms. To ensure resistance to bacteria on leather surfaces, protection solutions need to be developed, addressing both environmental issues and economic viability. In this work, chitosan nano/microparticles (CNP) and chitosan/silver nano/microstructures (CSNP), containing silver nanoparticles around 17 nm size, were incorporated into leather, obtained from the industrial process. Low loads of chitosan-based nano/microformulations, 0.1% mass ratio, resulted in total bacteria reduction (100%) after 2 h towards Gram-positive *Staphylococcus aureus*, both with CNP and CSNP coatings. Otherwise, comparable tests with the Gram-negative bacteria, *Klebsiella pneumoniae*, *Escherichia coli*, showed no significant improvement under the coating acidic conditions. The antimicrobial activity was evaluated by standard test methods: (1) inhibition halo and (2) dynamic contact conditions. The developed protection of leather either with CNP or CSNP is much higher than the one obtained with a simple chitosan solution.

## 1. Introduction

Currently, there is a great concern with hygiene, comfort and quality of footwear, which poses new challenges to the tanning industries. The contact of the user’s foot with the shoe provides a favorable environment (heat, humidity and nutrients present in the sweat) [1] for the development of bacteria and fungi, which cause a deterioration of the leathers, unpleasant odors, and is a focus of infections [2,3,4]. In short, preventing and reducing the growth of microorganisms is a very topical issue in the tanning industry [5].

Commercially, there are some options to combat/control the proliferation of microorganisms in these environments. They consist mainly of the use of antiperspirants and spray formulations containing antibacterial or antifungal substances for application on the foot and inside the shoe. Hygroscopic insoles can also be used to minimize the presence of moisture [6]. In tanneries, biocides are used during the leather making process to stop putrefaction before tanning but are seldom used after this point [1,7,8,9]. The scientific literature reports new options: (1) zinc oxide nanoparticles incorporation [10]; (2) the deposition of silver agglomerates on the leather surface [11]; (3) melamine-formaldehyde microcapsules containing tea tree oil incorporation [3]; (4) essential oils from plants, such as encapsulated eucalyptus, lavender and thyme treatment [9,12]; (5) polyurethane dispersions added with photoactive antimicrobial agents used in coating [13]; (6) chitosan solutions (acidic) [5]; and methacrylic acid-acrylamide-chitosan polymer coatings [14].

Chitosan, obtained from chitin [15,16], was chosen for the present study because it is a non-toxic, biocompatible, and biodegradable material [17,18,19]. It is a very attractive biopolymer with a rich biocide activity, due to its ability to form polyelectrolyte films.

Due to these unique characteristics, chitosan is considered a viable material for the development of new functional coatings with applications in many sensitive areas, including water treatment, an introduction in medical devices, medical implants and the textile industry [20,21,22,23]. Good antimicrobial activity in leather had also been described [5]. The incorporation of chitosan in leather is possible due to electrostatic interactions between the positive ionic chitosan (–NH_3_^+^), the negative leather proteins (–CO_2_^−^) and other negative species present, such as dyes and fats [5,24]. We envisage that the application of chitosan microformulations might result in a higher bactericidal activity than the simple chitosan solution reported in the literature [5]. A recent review by Yuan et al. fully covers this topic [25].

The more efficient antibacterial activity of chitosan nanoparticles compared to chitosan solution would be due to the larger surface contact area of nanoparticles with the environment, eventually containing bacterial species. Although the effect of nanoparticles in the human body and in nature has been recently questioned, polysaccharide-based nanoparticles are known to be environmentally friendly, biodegradable and stable in physiological conditions, with much fewer concerns related to toxicity. Chitosan-silver nanoparticles will be also made due to the well-known antimicrobial efficacy of silver, to eventually find a better solution than simple chitosan nanoparticles. Modified silver nanoparticles in combination with gallic acid in coating sheep leather, have before been demonstrated to bear excellent and long-term antibacterial activity [26].

The economic viability in terms of industrial purposes is a main point in the present investigation. The work addresses: (1) the synthesis and characterization of chitosan, and chitosan/silver nano/microparticles; (2) its incorporation in leather obtained from the final dyeing stage, provided by a local tannery (incorporations were performed at the laboratory level, mimicking drum industrial process); and (3) the evaluation of antimicrobial protection of the treated leather against Gram-negative and Gram-positive bacteria.

## 2. Materials and Methods

### 2.1. Preparation of the Chitosan Solution (CS)

High-density chitosan (90/200/A1), obtained from BioLog Heppe GmbH (Landsberg, Germany), corresponding to a degree of deacetylation of 90% and dynamic viscosity of 170 mPa.s, was used to prepare the solutions and the micro/nanoparticles. The chitosan solution (1%, *w*/*v*) was prepared by adding high-density chitosan to an aqueous solution of formic acid (2% *v*/*v*). The suspension was stirred at 50 °C until complete dissolution gave a very viscous solution (CS), which was stored at 10 °C for later use.

### 2.2. Preparation of Chitosan Nano/Microparticles (CNP)

The preparation of chitosan nano/microparticles was adapted from the procedure described by Qi et al. [27]. The pH of the prepared CS (1 L, 1% *w*/*v*) was set to values between 4.6 and 4.8, by the addition of 10 M NaOH aqueous solution. An aqueous sodium tripolyphosphate (TPP) solution (668 mL, 0.25% *w*/*v*) was added dropwise, and the mixture was maintained under vigorous magnetic stirring. After 1 h, 10 M NaOH aqueous solution was added until pH = 7. The suspension was filtered under vacuum and successively washed with water (2 L), giving a pasty solid. The solid was collected and lyophilized, yielding CNP as a white solid (13.8 g) in quantitative yield, based on the amounts of chitosan and TPP added.

### 2.3. Preparation of Chitosan and Silver Nano/Microparticles (CSNP)

The preparation of chitosan/silver nano/microparticles was performed in the suspension containing chitosan nano/microparticles [28]. For that, to the cloudy suspension of chitosan nanoparticles prepared in procedure 2.2, was added an aqueous solution of silver sulfate (1 L; 2 × 10^−4^ M). The reaction mixture was stirred for one hour. To the resulting suspension, a 1 M sodium borohydride solution, in a 0.3 M sodium hydroxide aqueous solution (175 mL) was added dropwise. A vigorous magnetic stirring was continued until the hydrogen evolution ceased (4 h). CSNPs were filtered and successively washed with water (2 L). A thick golden paste was lyophilized, giving CSNPs as a golden solid (12.5 g), corresponding to a quantitative yield, based on the amounts of chitosan and TPP added.

### 2.4. Incorporation of Chitosan Nano/Microparticles in Leather Samples

Leather samples provided by a local tanning industry consisted of several hides, from the same batch (raw material) collected at four different phases of the dyeing industrial process: pre-dyeing phase at pH = 5 (PD), dyeing phase at pH = 5 (D), greasing phase at pH = 5 (G), and the finishing process at pH = 3 (E). The hides were cut into 2 × 2 cm^2^ squares, and then the chitosan-based formulations were incorporated. A 2% formic acid aqueous solution containing each chitosan formulation, CNP 1% *w*/*w*, or CNP 0.1% *w*/*w*, or CSNP 0.1% *w*/*w*, or CSNP 0.01% *w*/*w*, or CSNP 0.001% *w*/*w* (*w*/*w* ratio corresponding to the weight of chitosan formulation/weight of wet leather) was prepared and kept under magnetic stirring at 60 °C until the chitosan formulation was completely dissolved. In parallel, chitosan solution (CS 1% *w*/*w*) was diluted accordingly, for comparison. The leather square sample was dipped in the aqueous mixture at 60 °C, 40 °C or 25 °C to cook, under rotational stirring for 20 min or for 1 h. A solution volume (mL) corresponding to two times the weight (g) of the leather sample was used. The sample was drained, ironed and left to dry in air for 72 h. Control tests were obtained in alignment with the different conditions stated ahead, but with no chitosan formulations added. 

### 2.5. Characterization of CNP and CSNP

#### 2.5.1. X-ray Diffraction (XRD) and UV/Vis Absorption Spectroscopy

X-ray diffraction (XRD) analyses were performed using a conventional PAN’alytical X’Pert PRO diffractometer (Malvern Panalytical Ltd., Malvern, UK), operating with CuK_α_ radiation, in a Bragg–Brentano configuration. The absorption spectra were recorded in a Shimadzu UV-3600 Plus UV-Vis-NIR spectrophotometer (Shimadzu Corporation, Kyoto, Japan). A 150 mm integrating sphere (ISR-1503), with three detectors, was used for reflectance measurements of coated and uncoated leather. 

#### 2.5.2. Scanning Electron Microscopy Images

Scanning electron microscopy (SEM) images were recorded using a scanning electron microscope FEI-Nova 200 NanoSEM (FEI Technologies, Inc., Hillsboro, OR, USA), operating in transmission mode (STEM), at SEMAT (Serviços de Caracterização de Materiais, Guimarães, Portugal). A drop of the nanoparticles’ dispersion was placed onto a Formvar grid, held by tweezers. After 20 s, almost all the solution was removed with filter paper and left dry. The processing of SEM images was performed using ImageJ software. The area of each particle allowed an estimation of its size. The resulting histograms were fitted to Gaussian distributions. 

#### 2.5.3. Size and Zeta-Potential Measurements

Hydrodynamic diameters and zeta-potential values were measured using a dynamic light scattering (DLS) equipment NANO ZS Malvern Zetasizer (Malvern Panalytical Ltd., Malvern, UK) at 25 °C, using a He-Ne laser of 632.8 nm and a detector angle of 173°. Five independent measurements were performed for each sample. Malvern dispersion technology software (DTS) was used with multiple narrow modes (high resolution) data processing.

### 2.6. Antibacterial Activity Tests

To assess antibacterial activity, two different biological tests were carried out: the standard method under dynamic contact conditions, according to ISO 16187-2013 [29] (quantitative) and an antimicrobial sensitivity test, the halo inhibition method (qualitative). The inoculum was prepared by adding in a sterile medium a portion of the bacterium *Staphylococcus aureus* ATCC 6538 (as representative of Gram-positive bacteria) and *Escherichia coli* 434 or *Klebsiella pneumoniae* ATCC 11296 (as representative of Gram-negative bacteria) to 40 mL of TSB (tryptic soy broth). The bacteria grew in an orbital incubator at 37 °C, with orbital shaking at 120 rpm, overnight (≈18 h). At the end of this time, the optical density of the inoculum (OD, λ = 620 nm) was measured, and the necessary dilution was made to obtain an optical density close to 0.086 for *E. coli*, 0.120 for *S. aureus* and 0.110 for *K. pneumoniae*, which corresponds to a cell concentration of approximately 1 × 10^8^ CFU/mL in each case.

#### 2.6.1. Standard Test Method under Dynamic Contact Conditions

The cell inoculum was prepared with NaCl 0.9% aqueous solution (50 mL) to obtain a bacterial concentration of 1.5–3.0 × 10^5^ CFU/mL for the different tests. After dipping the leather samples, a sample was collected at the initial time (T_0_), and the flasks were then placed in an incubator at 37 °C, under orbital shaking at 120 rpm. Cell samples were collected at different times to determine the bacterial concentration using the colony-forming unit (CFU) counting technique. After each sampling, the flask was placed again in the orbital incubator. The results of the colony count were converted to colony-forming units per milliliter (CFU/mL) and used to calculate the log reduction and the percentage of bacteria reduction. The control was carried out with an uncoated leather sample run in parallel. For each contact time, the percentage of reduction in the number of colonies was calculated according to the following equation:

(1) $$\mathrm{Reduction}\;\left(\%\right)\left(\mathrm{CFU}/\mathrm{mL}\right)=\frac{B-A}{B}\times 100$$

where B is the number of colonies counted in the sample’s control test, and A is the number of colonies counted in tests with the leather samples containing chitosan formulations.

#### 2.6.2. Halo Inhibition Method

In the antimicrobial susceptibility test, on a Petri dish containing plate count agar (PCA) 15 mL, was spread 0.1 mL of the bacterial solution (1 × 10^8^ CFU/mL) over the dry agar. The prepared dried leather samples (control and coated samples with chitosan formulations) were placed in the center of each Petri dish on the agar surface and were incubated at 37 °C. Measurements of the inhibition zone were taken after 8 h, 24 h and 48 h incubation time.

## 3. Results and Discussion

### 3.1. Synthesis of Chitosan Nano/Microstructures (CNP)

The chitosan nano/microstructures were synthesized by the ionotropic gelation method. Ionic interactions between TPP anions and ammonium groups (cations) of the protonated chitosan allow polymeric formation by inter- and intramolecular crosslinks, leading to the formation of CNP compact structures [27,30,31]. The synthetic procedure includes a significant yield improvement, achieved by replacing the usual centrifugation by precipitation obtained by adjusting pH to 7 with 10 M NaOH solution. The suspension was filtered under vacuum, and the obtained cake was washed thoroughly to give a soggy solid, which after lyophilizing led to CNP in a quantitative yield.

CSNP are obtained from the CNP preparation at the precipitation stage when a gelatinous suspension is formed. The addition of an aqueous solution of silver sulfate to the previous mixture allowed the formation of electrostatic interactions between silver ions (Ag^+^) and the chitosan polymer, through its electron-rich hydroxyl groups. The metal cations adsorbed on the outer surface of the CNP, are afterwards reduced to metallic silver (Ag^0^) within the chitosan matrix, by the addition of sodium borohydride [32,33]. This is an optimized procedure, as the silver cations are adsorbed to the already formed CNP, increasing the active surface area. The polymeric structure of chitosan enables Ag^0^ stabilization [34]. The production of the CSNP was also optimized, by replacing the centrifugation with simple vacuum filtration. The wet solid cake obtained was washed thoroughly with water and lyophilized to give a golden solid in quantitative yield. 

### 3.2. Chitosan Nano/Microstructures Characterization

**Figure 1:**
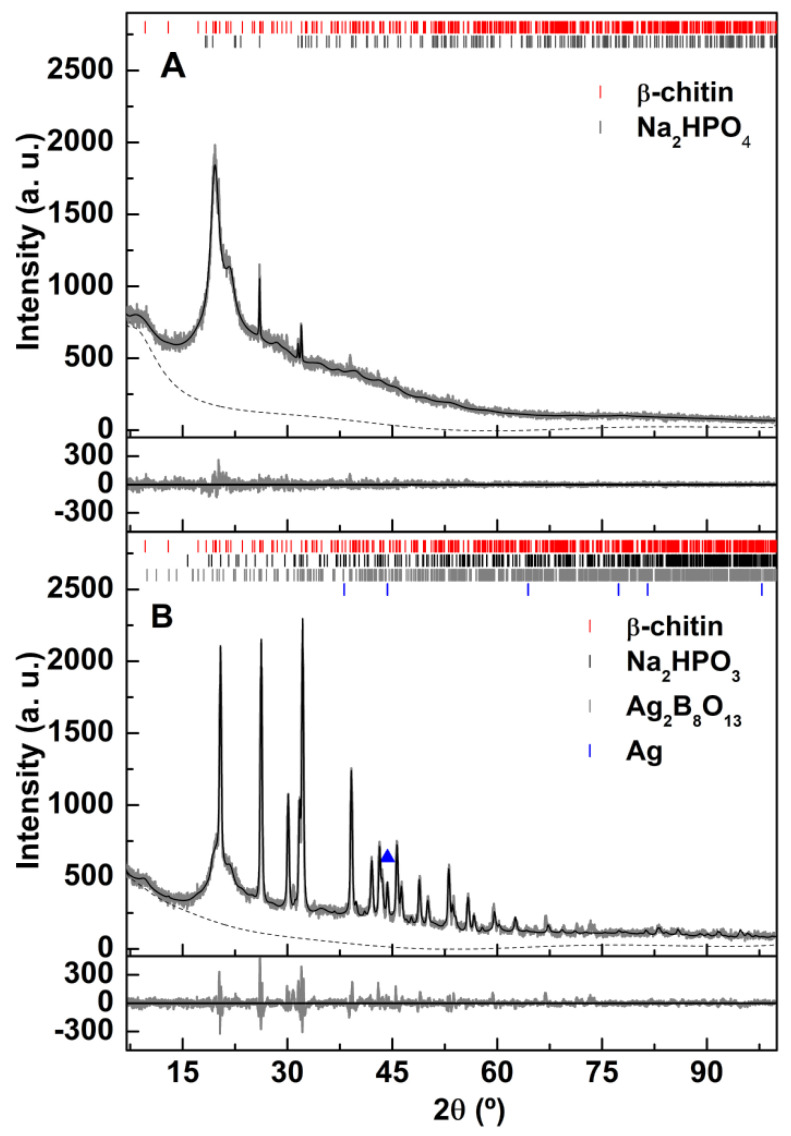
XRD diffractograms of (**A**): CNP; (**B**): CSNP. The silver diffraction peak is labeled with a triangle.

XRD measurements of chitosan-derived samples depend on the degree of acetylation and on the underlying structure, which can be either α or β [35,36]. The preparation method of chitosan nanoparticles frequently involves crosslinking of chitosan polymeric chains with TPP anions, resulting in an amorphous structure [27], which can, however, also show some local organization, as evidenced by the appearance of some diffraction peaks [37]. Figure 1 evidences that the prepared chitosan nanoparticles are composed of β-chitosan with some sharp peaks, similar to the results reported by Gokila et al. [37]. For the analysis of XRD diffractograms, *Profex* software [38] was used, which is based on *BGMN* [39] Rietveld calculations. The chitosan phase was analyzed using β-chitin CIF file nr. 1501776 (space group P2_1_) and allowing two different peak widths, one reflecting the amorphous nature and the other with the possible localized ordering of the chitosan chains. A good fit was obtained, with χ^2^ =1.34 and R_P_ = 4.59%. The observed sharp peaks are compatible with the presence of a Na_2_HPO_4_ phase (nahpoite, CIF file nr. 9012339, space group P2_1_/m), with an estimated size of 193 nm, using the implementation of size broadening effects on *BGMN* [39].

The incorporation of silver through reduction with NaBH_4_ results in the appearance of several other diffraction peaks (Figure 1B). They were successfully assigned to sodium hydrogen phosphite (Na_2_HPO_3_, mp-556699 [40], space group P2_1_/c), silver borate (Ag_2_B_8_O_13_, mp-554873 [41], space group P2_1_/c), and metallic silver (CIF file nr. 9011608, space group Fm$\bar{3}$m). A Rietveld analysis resulted in a reasonable fit, with χ^2^ =3.44 and R_P_ = 8.27%, with sizes of 37 nm, 38 nm and 55 nm, respectively. For silver, only the reflection corresponding to (2 0 0) is observed. Table S1 in Supplementary Materials shows the main results of the Rietveld analysis. 

The diffractograms of both samples were obtained after lyophilization. This process involves an initial freezing of water in the sample, originating very high concentrations of solutes and particles within the aqueous phase that have not solidified [42]. This usually induces particle aggregation and salts crystallization, as well as stress at the particles’ surface. The appearance of silver borate might thus result from the crystallization of surface adsorbed borates, from the NaBH_4_ reduction process, with silver ions that are either co-adsorbed or result from oxidation of silver under the stressful conditions of the lyophilization process. This procedure also explains the presence of the sharp diffraction peaks corresponding to the sodium hydrogen phosphate phase, as TPP was used in high excess during the chitosan nanoparticles synthesis. The strong reduction conditions resulting from the use of NaBH_4_ justify the appearance of the sodium hydrogen phosphite phase in the chitosan-silver sample.

The UV/Vis absorption spectrum of Ag-doped chitosan nanostructures evidences the presence of the silver plasmon band around 400 nm (Figure S1 in Supplementary Material). The position of the silver plasmon band (Figure S1) depends on the size, shape and refraction index of the medium surrounding the silver nanoparticle [43]. Considering silver nanospheres in water, for which citrate was used as a stabilizing agent [41], the plasmon band for 10 nm size nanoparticles appears at 398 nm [44]. Here, a slightly larger size is expected from the plasmon band maximum at 404 nm (Figure S1), considering that the silver nanoparticles are embedded in chitosan dispersions, which the refractive index (around 1.34–1.35 for visible light [45]) is similar to that of citric acid (*n* = 1.34–1.36 for aqueous solutions at room temperature, depending on the concentration [46]). 

**Figure 2:**
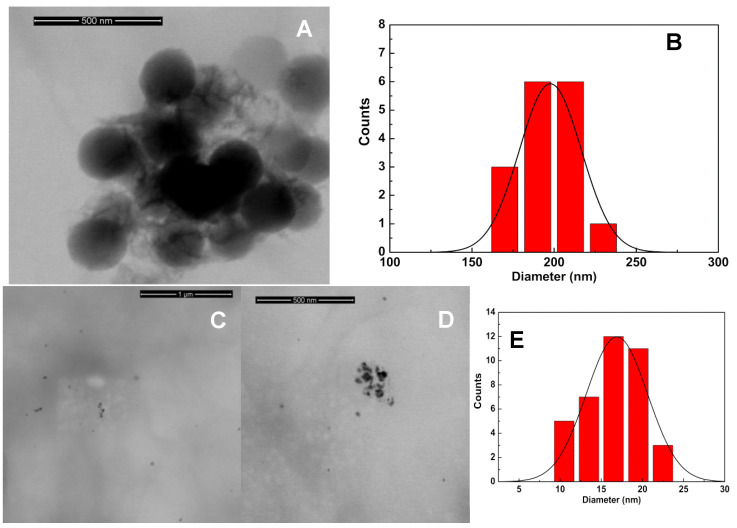
(**A**). SEM image of chitosan nanostructures. (**B**). Size histogram of the chitosan nanoparticles and fitting to a Gaussian distribution. (**C**,**D**). SEM images of silver nanoparticles embedded in chitosan fibrous structures at different magnifications. (**E**). Size histogram of nanoparticles in the image (**D**) and fitting to a Gaussian distribution.

SEM measurements (obtained in transmission mode) evidence the presence of spherical nanoparticles with a uniform size around 200 nm (Figure 2A), together with chitosan fibrous microstructures. The size histogram (Figure 2B) allows the calculation of an average diameter of 198 ± 18 nm for the observed nanoparticles. Small silver nanoparticles embedded in extended fibrous chitosan microstructures can be observed (Figure 2C,D), with some aggregates. The size histogram (Figure 2E) reveals nanoparticles of 16.9 ± 3.7 nm, following what was predicted from the absorption spectrum. The higher size of silver nanoparticles determined by XRD measurements might be due to aggregation effects induced by the lyophilization process. 

**Figure 3:**
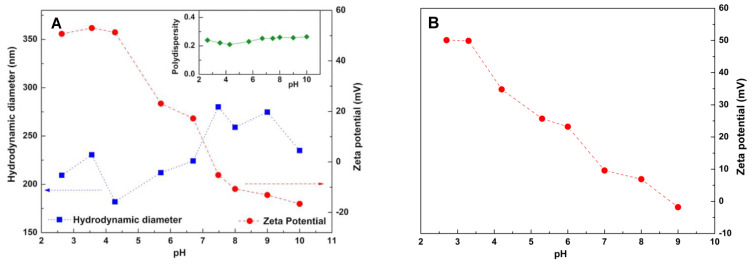
(**A**). Hydrodynamic diameter and zeta-potential values of chitosan nanostructures, at different pH values. Inset: Polydispersity index. (**B**). Zeta-potential values of chitosan/silver nanocomposites in aqueous solution at different pH values.

Zeta-potential is an important parameter for evaluating the stability of nanosystems in aqueous suspension. Zeta-potential reflects surface charge, which influences stability/aggregation in suspension, due to electrostatic repulsion between particles. Moreover, surface charge influences the interaction between nanostructures and the bacteria membrane, which is usually negative [27]. Therefore, the effect of pH variation on the surface charge of chitosan-based nanostructures (with and without silver nanoparticles) was investigated by zeta-potential measurements, determining also the hydrodynamic diameter of chitosan nanostructures (Figure 3). The hydrodynamic diameter varies in the range of 180–280 nm. A reasonable polydispersity (PDI) was obtained (around 0.21–0.25) despite, in some cases, PDI being near 0.3, revealing more polydisperse structures.

Chitosan nanostructures show zeta-potential values ranging from +57 mV to −18 mV (Figure 3A). At low pH values, chitosan nanoparticles exhibit a high positive surface charge, due to the protonation of amino groups. At high pH values, the surface charge is negative. It is worth noting that chitosan is soluble in dilute aqueous acidic conditions with pH below 6.0, which transforms glucosamine units into soluble ammonium forms (R-NH_3_^+^) [47,48]. Thus, the amino groups get protonated, leading to the formation of a cationic polyelectrolyte. When pH is above 6.0, these amino groups become deprotonated [49], the nanostructures becoming negatively charged at pH higher than 7. At slightly negative zeta-potential values (pH between 7.4 and 9), a tendency to larger sizes (higher aggregation) is inferred. Considering chitosan/silver nanocomposites, zeta-potential with pH variation was also determined, showing a decrease in absolute values relative to neat chitosan (Figure 3B).

### 3.3. Incorporation of Chitosan-Based Nano/Microformulations into the Leather

The method used to incorporate chitosan-based nano/microformulations in leather samples simulates drum processing within the industrial process. Leather samples from the four different stages of the dyeing process were included in this study. Incorporation of chitosan-based solutions CS, CNP and CSNP to the leather samples tentatively used the remaining bath from the industrial dyeing process. However, chitosan precipitated out as soon as a chitosan solution (CS, CNP, and CSNP) was added to the bath. Precipitation is possibly due to the collapse of the positive chitosan matrix with remaining anionic dyes in the bath. As so, an aqueous solution of 2% formic acid was used in the incorporation step instead of the industrial bath.

**Figure 4:**
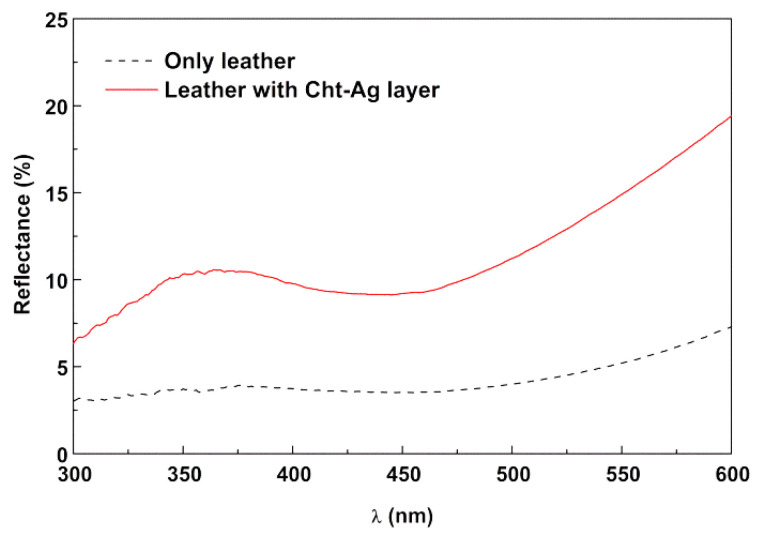
Total reflectance spectra of uncoated leather and leather coated with CSNP dispersion in 2% formic acid solution.

Evidence of leather coating with CSNP nanostructures is obtained by reflectance measurements. CSNP aqueous dispersions are very light yellowish, as a result of white chitosan and silver absorption in the violet/blue region of the spectrum. When covered by CSNP nanostructures dispersion (in 2% formic acid in water), the leather exhibits an increase in reflectance (lighter surface) and a valley in the region of 400–450 nm due to silver absorption (Figure 4). The increase of reflectance above 600 nm is due to the brown color of the leather sample used. For a suitable comparison, the uncoated leather was also immersed in a 2% formic acid solution.

### 3.4. Antimicrobial Assays

In this work, two different biological tests were carried out: the antimicrobial susceptibility test or inhibition halo (qualitative), and the standard test under dynamic contact conditions, according to ISO 16187-2013 (quantitative) [29].

#### 3.4.1. Antimicrobial Susceptibility Tests

In the antimicrobial susceptibility tests, the inhibition zone is measured; the diameter of this zone reflects the magnitude of the microorganisms’ susceptibility to the antimicrobial agent used. Thus, bacterial strains susceptible to the prepared chitosan nano/microformulations should exhibit a larger diameter of inhibition, while more resistant strains will have a smaller diameter of inhibition. Initially, a survey was carried out with samples from different stages of the tanning industrial dyeing process: pre-dyeing (PD), dyeing (D), greasing (G) and end of the process (E). The purpose of this series of tests is to evaluate the biological activity inherent to the sample obtained from the various industrial phases. Table 1 shows the results of the antimicrobial tests of the halo against *S. aureus* in leather samples from the stages above (PD, D, G, E), either with dry (d) or wet (w) samples and with or without formic acid (FA). Formic acid was added considering that, at the end of the retanning industrial process, it is used to assist the fixing of dyes and tanning greases.

A quick survey across Table 1 evidences that only samples with formic acid show antimicrobial activity. Samples without acid treatment show no activity at all. Additionally, wet samples with acid display higher activity than dry samples with acid, probably due to an increase in the diffusion ability. Accordingly, it was decided to choose dry samples in the inhibition evaluating tests.

Leather samples for chitosan incorporation were selected from the dyeing phases D and E. A direct incorporation of the chitosan formulation at the dyeing level of the industrial process was tried, but chitosan precipitates out of the industrial treating bath. In fact, implementation of the cure at this stage would recommend an entirely new bath for such a purpose. On the other hand, an application of chitosan nano/microformulations at the ending level has no implication with the established industrial process, because it consists precisely in addition of formic acid, as it occurs with the dissolution of chitosan materials needed for coating applications. Thus, an implementation of the antimicrobial chitosan treatment at this point of the industrial process would be highly desirable.

**Figure 5:**
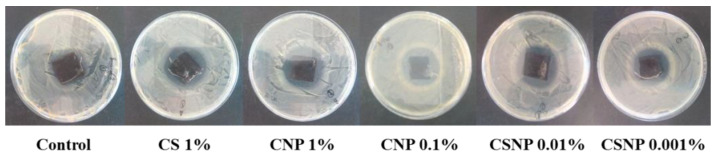
Halo tests in leather samples from the dyeing phase (D) against *S. aureus*.

Table 2 shows the measurement of halos in antimicrobial tests against *S. aureus* bacteria in leather samples obtained from stage D in the industrial process, after the incorporation of chitosan solution CS 1%, and chitosan nano/microformulations (CNP 1%, CNP 0.1%, CSNP 0.01%, and CSNP 0.001%). The treatment bath for every sample consisted of cooking at 60 °C for 1 h, under rotational stirring, with a mixture of the antimicrobial agent, formic acid and water, according to Section 2.4. The control test was placed in parallel conditions, without chitosan. Inhibition halos (in mm) showed larger than controls in every case tested (Figure 5).

Table 2 shows that chitosan micro/nanostructures coatings are much more active than chitosan in solution. The best result was obtained for CNP 1%. Concentration 0.1% or even 0.01% exhibits better inhibitory activity than simple chitosan: an average inhibition ratio of 12 mm in CSNP 0.1%, 10 mm in CSNP 0.01%, and 8 mm in CSNP 0.001% after 24 h time incubation. Such results enable us to think that micro/nanostructures based on chitosan may turn out to be economically feasible for application in the leather industry.

The bacterial inhibition of *E. coli* and *K. pneumoniae* bacteria was also studied for the dyeing industrial D stage. The inhibition halos for these bacteria are much larger than for *S. aureus* but are substantially identical to the control. The results obtained for *E. coli* and K. *pneumoniae* indicate that the industrial process might already create good protection against Gram-negative bacteria. The inhibition area in controls is around 20 mm for *E. coli*, and 35 mm for *K. pneumoniae*, not comparable to tests in *S. aureus*.

Table 3 shows the inhibition halos obtained for the leather samples at the end of the industrial process E against *S. aureus*. Different incorporation (cooking) times and temperatures were tested, to find differences in inhibitory activity. Close results of aleatory tests were obtained for different formulations/concentrations, showing the differences to be meaningless in terms of the protection obtained. Treatment with the nano/microformulations CNP 0.1% and CSNP 0.1%, showed very good results, which were slightly better in the case CSNP 0.1%. Treatment with CNP 0.01% is less effective. CNP 1% was not considered because of economic impracticability in view of industrial purposes. Both CSN and CSNP at 0.1% concentration seem to be economically viable.

Parallel experiments were run for *K. pneumonia* (not shown). The halo shown was very large, and comparable to controls, in line with the previous results obtained for the dyeing phase (D). The antibacterial effect of chitosan micro/nanostructures is offset by the components present in leather in virtue of the industrial process itself in the case of *K. pneumonia*.

In conclusion, the inhibition zone tests (*S. aureus*) proved to be useful to verify (qualitatively) the increase in the antimicrobial effect of leathers treated with chitosan formulations when compared to the control sample. These results served as a basis for choosing the chitosan nano/microformulations. Significantly, a clear antimicrobial activity improvement was observed with the application of CNP and CSNP in respect to the effect of chitosan solution application, reported in the literature [5,50].

#### 3.4.2. Antimicrobial Test of DYNAMIC Contact

In the antimicrobial dynamic contact test, the leather coupon was immersed in a cell suspension, and samples were then collected to quantify the number of microorganisms present in solution. Table 4 records the bacterial inhibition (in%) of the antimicrobial dynamic contact tests in leather samples from the dyeing process (D) with different chitosan formulations/concentrations against *S. aureus*, after 2 h and 4 h (T2 and T4). Leather samples were treated with chitosan solution CS 1%, or nano/microformulations: CNP 1%, CNP 0.1%, CSNP 0.01% and CSNP 0.001%. All samples tested (control included) were cooked at 60 °C for 1 h.

A greater capacity for bacterial inhibition, close to 100%, was found in samples treated with CNP 1% and 0.1%. These results begin to build up after 2 h (T2) and are 100% after 4 h (T4). Leather samples containing CNP or CSNP treated both with 0.01% and 0.001% did not show good results in this antimicrobial test, in contrast to the halo test. After 8 h, both control and leather samples treated with chitosan formulations showed the total bactericidal effect. This increased activity over time is due to the industrial antimicrobial agents present in the leather samples (chromium, dyes, greases, etc.) that diffuse to the solution and kill bacteria.

Table 5 shows the results of bacterial inhibition (in%) in the dynamic contact antimicrobial tests against *S. aureus* in leather samples obtained in industrial phase E, incorporating chitosan nano/microformulations (CNP 0.1%, CSNP 0.1% and 0.01%). All the tested samples (control included) were treated as follows: they were placed in water, containing chitosan or chitosan formulations dissolved in formic acid, cooked for 20 min at 25 °C, under mechanical stirring. The most economically viable nano/microformulation solutions were used in these tests: CNP 0.1%, CSNP 0.1% and 0.01%. The samples containing the formulations CNP 0.1% and CSNP 0.1% provided better bacterial inhibition results, very close to 100%, within 2 h. The results of the antibacterial effect in samples with CSNP 0.01% after 2 h or even 4 h, compared to the control, are low. Again, after 8 h, the antimicrobial effect was total, both in the controls and in the samples with the antimicrobial agent. The dynamic contact antimicrobial test was also performed for *K. pneumoniae* in leather samples in industrial phase E, incorporating the chitosan nano/microformulations (CNP 0.1%, CSNP 0.1%, and 0.01%) under the same conditions. The results obtained revealed a total bactericidal effect after 2 h, both in leather samples treated with the antimicrobial agent and in control.

In conclusion, dynamic contact tests revealed a good antibacterial behavior of the CNP and CSNP. The application of these micro/nanoformulations in a 0.1% concentration showed an antibacterial effect identical to simple chitosan solution in 1% concentration, reported in the literature [50]. Such an improvement in the bactericidal activity observed in the chitosan nano/microformulations might be found economically viable for future application in the tanning industry [5,50].

## 4. Conclusions

In the present work, CNP and CSNP were obtained in quantitative yields and simplified methodology. The incorporation of chitosan nano/microformulations was performed in leather obtained from the industrial dyeing phase (three sub-phases: D, G, E) and their antimicrobial activity was evaluated. Results are best at the ending E dyeing phase. The antimicrobial inhibition results obtained for Gram-positive bacteria (*S. aureus*) are promising in 0.1% chitosan nano/microparticles concentrations, both for CNP and CSNP, resulting in antibacterial protection identical to the one conferred by the 1% chitosan solution. Tests against Gram-negative bacteria, *E. coli* and *K. pneumonia*, show that efficient protection against these bacteria might be already offered by the tanning industrial process itself, in which formic acid is used for fixing dyes (large halos are observed either in CNP-coated leather samples and in leather samples treated with 2% formic acid). Nevertheless, *S. aureus* is a major pathogenic bacterium capable of causing skin foot infections and is quite efficiently addressed by chitosan nano/microstructures studied in this work. In short, a drum-type application of CNP and CSNP in a 0.1% (*w*/*w*) to leathers at very mild conditions, 25 °C for 20 min. in water, compatible with the fixing final industrial step confers extra protection against Gram-positive *S. aureus*, and its application to the leather industry might be economically viable, reducing protection step cost relative to the state-of-the art (1% chitosan solution). The cost will be reduced *c.a.* 10 times for 0.1% CNP, and *c.a.* 8 times for 0.1% CSNP.

## Figures and Tables

**Table 1:** Inhibition halos (in mm) for different leather samples (dry (d) or wet (w)) with formic acid (FA) or without formic acid tested against *S. aureus*. Samples were obtained from the industrial process (pre-dyeing (PD), dyeing (D), greasing (G), and at the end (E)), cooked for 1 h at 60 °C with different contact times (T8, T24, and T48). The measure of the halo (in mm) results from the subtraction of the size of the leather square (20 mm × 20 mm) to the total halo.

	Tests with *S. aureus* Inhibition Halo (mm)		
	T8	T24	T48
PD (d)	0	0	0
PD + FA (d)	8.8	6.8	1.8
PD (w)	0	0	0
PD + FA (w)	19.5	10	7.5
D (d)	0	0	0
D + FA (d) *	8.8	4	0
D (w)	0	0	0
D + FA (w)	19	14.8	8.8
G (d)	0	0	0
G + FA (d)	16.8	4.3	3
G (w)	0	0	0
G + FA (w)	18	9.5	8.3
E (d)	0	0	0
E + FA (d) *	16.3	7.5	1.8
E (w)	0	0	0
E + FA (w)	24.8	17	14

* Best samples, to be selected in subsequent antimicrobial inhibition tests.

**Table 2:** Inhibition halo (mm) of leather samples obtained from dyeing industrial process D after treatment with CS 1%, CNP 1% or 0.1%, CSNP 0.01% or 0.001%, against *S. aureus.* The measurement of the halo presented is obtained by subtracting the control’s halo to the measure of the sample’s halo under study.

	Inhibition Halo (mm)	
	T24	T48
CS 1%	9.2	6.7
CNP 1%	15.3	9.8
CNP 0.1%	11.8	7.8
CSNP 0.01%	10.2	7.3
CSNP 0.001%	8.0	5.2

**Table 3:** Inhibition halos (mm) of the antimicrobial tests in leather samples at phase E treated with chitosan nano/microformulations: CNP 0.1%, CSNP 0.1%, and CSNP 0.01%, against S. aureus. Incorporations were run at different temperatures and times. The measure of the inhibition halo corresponds to the halo after subtracting the control (leather).

		Inhibition Halo *S. aureus* (mm)	
		T24	T48
CNP 0.1%	25 °C; 20 min	6.0	5.5
	25 °C; 1 h	7.0	5.8
	40 °C; 20 min	6.0	5.2
	40 °C; 1 h	4.2	4.8
CSNP 0.1%	25 °C; 20 min	6.8	6.2
	25 °C; 1 h	7.2	6.2
	40 °C; 20 min	6.7	6.0
	40 °C; 1 h	5.2	5.3
CSNP 0.01%	25 °C; 20 min	2.0	3.3
	25 °C; 1 h	3.0	3.0
	40 °C; 20 min	3.2	3.2
	40 °C; 1 h	1.8	2.7

**Table 4:** Inhibition values (in%) obtained in the dynamic contact tests against S. aureus in leather samples from the dyeing process (D) treated with chitosan CS 1%, or chitosan nano/microformulations (CNP 1% and 0.1%, and CSNP 0.01% and 0.001%).

	Bacterial Inhibition (%) *S. aureus*	
	T2	T4
CS 1%	95 *	98 *
CNP 1%	98 *	100 *
CNP 0.1%	88 *	100 *
CSNP 0.01%	33 *	37.8 *
CSNP 0.001%	1 *	27 *

* Bacterial reduction when compared with the control.

**Table 5:** Bacterial inhibition (in%) obtained in dynamic contact antimicrobial tests of leather samples obtained from the industrial phase E containing nano/microformulations of chitosan (CNP 0.1%, CSNP 0.1% and 0.01%) against *S. aureus*.

	Bacterial Inhibition (%) *S. aureus*	
	T2	T4
CNP 0.1%	100 *	100 *
CSNP 0.1%	100 *	100 *
CSNP 0.01%	24 *	46 *

* Bacterial reduction compared to control.

## Data Availability

Data sharing is not applicable for this paper.

## Supplementary Materials

The following supporting information can be downloaded at: https://www.mdpi.com/article/10.3390/ma15051750/s1, Figure S1: UV/Visible absorption spectrum of silver nanoparticles in chitosan nanostructures; Table S1: Results of Rietveld analysis.
