# Supplementary material for: Chitosan Nano/Microformulations for Antimicrobial Protection of Leather with a Potential Impact in Tanning Industry
Source: Materials (Basel). 2022 Feb 25;15(5):1750. doi: 10.3390/ma15051750 (PMC8911499; doi:10.3390/ma15051750)
Supplement: Supplementary file 1 [file materials-15-01750-s001.zip › materials-1591899-supplementary.pdf]

materials

MDPI

Article

Chitosan Nano/Microformulations for Antimicrobial Protection of Leather with a Potential Impact in Tanning Industry

David S. Freitas [1], Pilar Teixeira [2,3], Inês B. Pinheiro [1], Elisabete M. S. Castanheira [4,5], Paulo J. G. Coutinho [4,5,*] and Maria J. Alves [1,*]

[1] Centre of Chemistry and Department of Chemistry, University of Minho, Campus de Gualtar, 4710-057 Braga, Portugal; davidsfreitas@ceb.uminho.pt (D.S.F.); inespinheiro@bio.uminho.pt (I.B.P.)
[2] CEB—Centre of Biological Engineering, University of Minho, Campus de Gualtar, 4710-057 Braga, Portugal; pilar@ceb.uminho.pt
[3] LABBELS—Associate Laboratory, 4800-122 Braga, Guimarães, Portugal
[4] Centre of Physics of Minho and Porto Universities (CF-UM-UP), University of Minho, Campus de Gualtar, 4710-057 Braga, Guimarães, Portugal; ecoutinho@fisica.uminho.pt
[5] LaPMET—Laboratory of Physics for Materials and Emergent Technologies, 4800-122 Braga, Guimarães, Portugal
* Correspondence: pcoutinho@fisica.uminho.pt (P.J.G.C.); mja@quimica.uminho.pt (M.J.A.)

Citation: Freitas, D.S.; Teixeira, P.; Pinheiro, I.B.; Castanheira, E.M.S.; Coutinho, P.J.G., Alves M.J. Chitosan Nano/Microformulations for Antimicrobial Protection of Leather with a Potential Impact in Tanning Industry. *Materials* **2022**, *15*, 1750. https://doi.org/10.3390/ma15051750

Academic Editor: Andrzej Dziedzic

Received: 26 January 2022
Accepted: 22 February 2022
Published: 25 February 2022

Publisher's Note: MDPI stays neutral with regard to jurisdictional claims in published maps and institutional affiliations.

Copyright: © 2022 by the authors. Licensee MDPI, Basel, Switzerland. This article is an open access article distributed under the terms and conditions of the Creative Commons Attribution (CC BY) license (https://creativecommons.org/licenses/by/4.0/).

Supplementary Material

Results of Rietveld analysis of XRD data

Table S1. Results of Rietveld analysis.

Sample	Phase	Space Group	Size (nm)	a (Å)<br>b (Å)<br>c (Å)	α<br>β<br>γ	χ^2	R_P
CNP	β-chitin	$P2_1$	---	4.8<br>9.2<br>10.2	90<br>90<br>97	1.34	4.59
	Na_2HPO_4	$P2_1/m$	193	5.45<br>6.84<br>5.40	90<br>117.0<br>90		
CSNP	β-chitin	$P2_1$	---	4.8 (*)<br>9.2 (*)<br>10.2 (*)	90<br>90<br>97 (*)	3.44	8.27
	Na_2HPO_3	$P2_1/c$	37	5.10<br>7.01<br>10.4	90<br>114.8<br>90		
	$Ag_2B_8O_{13}$	$P2_1/c$	38	6.78<br>8.78<br>17.8	90<br>89.9<br>90		
	Ag	$Fm\bar{3}m$	55	4.08<br>4.08<br>4.08	90<br>90<br>90		

(*) fixed value.

UV/Vis absorption spectrum of Ag-doped chitosan nanostructures

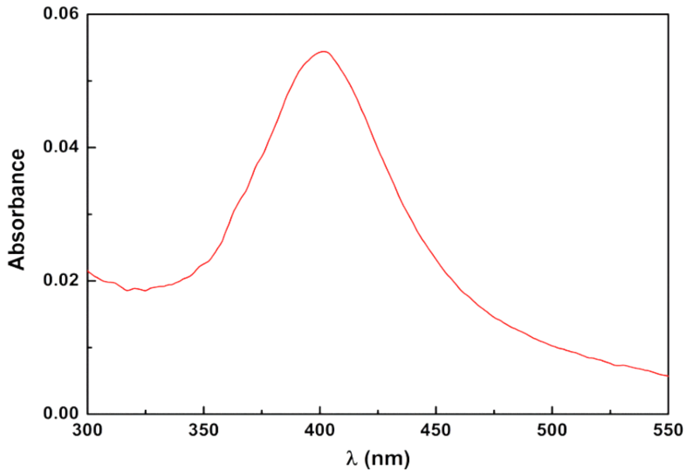

Figure S1. UV/Visible absorption spectrum of silver nanoparticles in chitosan nanostructures.
